# Fiber-tract localized diffusion coefficients highlight patterns of white matter disruption induced by proximity to glioma

**DOI:** 10.1371/journal.pone.0225323

**Published:** 2019-11-21

**Authors:** Shawn D’Souza, D. Ryan Ormond, Jamie Costabile, John A. Thompson

**Affiliations:** 1 Department of Molecular Biology, University of Colorado, Boulder, CO, United States of America; 2 Department of Neurosurgery, University of Colorado Anschutz Medical Campus, Aurora, CO, United States of America; 3 Department of Neurology, University of Colorado Anschutz Medical Campus, Aurora, CO, United States of America; George Washington University, UNITED STATES

## Abstract

Gliomas account for 26.5% of all primary central nervous system tumors. Recent studies have used diffusion tensor imaging (DTI) to extract white matter fibers and the diffusion coefficients derived from MR processing to provide useful, non-invasive insights into the extent of tumor invasion, axonal integrity, and gross differentiation of glioma from metastasis. Here, we extend this work by examining whether a tract-based analysis can improve non-invasive localization of tumor impact on white matter integrity. This study retrospectively analyzed preoperative magnetic resonance sequences highlighting contrast enhancement and DTI scans of 13 subjects that were biopsy-confirmed to have either high or low-grade glioma. We reconstructed the corticospinal tract and superior longitudinal fasciculus by applying atlas-based regions of interest to fibers derived from whole-brain deterministic streamline tractography. Within-subject comparison of hemispheric diffusion coefficients (e.g., fractional anisotropy and mean diffusivity) indicated higher levels of white matter degradation in the ipsilesional hemisphere. Novel application of along-tract analyses revealed that tracts traversing the tumor region showed significant white matter degradation compared to the contralesional hemisphere and ipsilesional tracts displaced by the tumor.

## Introduction

Gliomas are the most prevalent form of intrinsic brain tumor [1] originating from neural tissue composed primarily of astrocytes, oligodendrocytes, and ependymal cells [2] with varying symptoms depending on the extent of invasion and location in the brain [3,4]. Surgical resection of the tumor area remains a mainstay of conventional therapy, and extent of resection is a well-established predictor of patient survival [5–7]. Despite recent advances in surgical oncology, predictive biomarkers of tumor progression, and recurrence based on in vivo measures of microstructural change are still lacking, which could impact surgical decision-making.

Over the last 15 years, white matter fiber tracking, using diffusion tensor imaging (DTI) has been investigated in different aspects of the surgical management of gliomas [8]. DTI is an *in vivo* neuroimaging technique that measures anisotropic water diffusion to extract white matter tractography [9]. Thus far, DTI has been utilized as a structural tool to improve pre- and intraoperative resection technique and preserve postoperative functionality. Preoperative imaging locates eloquent tracts at risk of damage during resection and confirms tumor regions of excision and avoidance [10]. Preoperative imaging has also been used as a predictive tool to assess postresection morbidity and mortality [11,12]. Intraoperative use allows for correction of brain shift and increases the spatial resolution of functional brain mapping techniques, such as direct electrical stimulation [13,14].

In addition to the use of DTI as a structural tool, it has also been applied to indirectly and quantitatively measure the microstructural integrity of white matter. Four diffusion coefficients are produced from DTI analysis: axial diffusivity (AD), radial diffusivity (RD), mean diffusivity (MD), and fractional anisotropy (FA). FA and MD have traditionally been used to evaluate overall white matter health [15]. Though FA has shown high sensitivity to changes in white matter water diffusion in the context of neuropathologies, such as multiple sclerosis, schizophrenia, and Alzheimer’s disease, it cannot differentiate between specific types of neural injury (e.g., demyelination, axonal injury, inflammation) [15–20]. Similarly, elevated MD has consistently been observed in pathologies causing edema, inflammation, and necrosis [15]. To specify neural injury and white matter microstructural integrity, recent studies have begun incorporating AD and RD analyses. Early studies utilizing mice models found these measurements can differentiate between pathological demyelination or axonal injury, with an inverse relation between RD and myelination status and an inverse relation between AD and axonal degradation [21,22]. This study aims to further support the use of DTI as a pathophysiological tool by analyzing how glioma proximity affects the microstructural integrity of major white matter pathways.

In the present study, we used a novel application of along-tract-analysis (ATA), which allowed us to address how tumor impact varies with distance from tumor. ATA normalizes tract length across subjects, accounting for patient-specific neural anatomy [23,24]. We applied ATA to two major white matter pathways: Corticospinal Tract (CST), critical for conduction of voluntary movement from upper to lower motor neurons [25,26], and Superior Longitudinal Fasciculus (SLF), critical for motor coordination and speech function [27,28]. The CST and SLF were chosen based on anatomy and significance in glioma resection cases [27,29,30].

A within-in subject analysis was conducted on tumor-present and tumor-free hemispheres. Gross tractographic analysis at the level of the whole hemisphere indicated higher white matter degradation in the ipsilesional hemisphere compared to the contralesional hemisphere, confirming our previous findings [24]. Application of ATA to the CST and SLF highlighted regions of localized white matter degradation, across subjects, adjacent to or traversing the tumor area.

## Methods

### Subject demographics

All procedures and protocols for this study were reviewed and approved by the Colorado Multi-Institutional Review Board (COMIRB 17–1136) and followed in accordance with the relevant guidelines and regulations. Subjects included in this study were patients undergoing resective surgery, from January to December 2016 at the University of Colorado Hospital, to remove an intracranial tumor classified by histopathology as glioma requiring functional imaging due to localization in or near language or motor cortex. Eight patients were male (62%) and the average age was 40 years (range: 20–73). Data were collected retrospectively from patient chart review through the application of a consent exempt IRB protocol wherein only clinical data were reviewed after deidentification by a member of the study team. Initial patient population (n = 16) was screened before analysis. Two cases were removed due to tumor infiltration of the contralateral hemisphere. One case was removed due to the tumor being located within the ventricle, which prevented any tracts from running through the tumor region of interest. After screening, the glioma cases (n = 13) included in this study were a heterogeneous group of both high and low-grade tumors. Data represent a subset of patient data previously analyzed in Ormond et al., 2017.

### Imaging sequence parameters

All images were obtained using a 3.0-T whole-body MR imager (Signa HDx; GE Medical Systems, Milwaukee, Wisconsin, USA) using single-shot echo-planar imaging. Acquisition times were approximately 3.5 minutes for T2-weighted images (T2w) and 9 minutes for DT images. For T2w, TE = 102.96ms, TR = 5781ms, and flip angle = 90°. Data were recorded with a 512 x 512 spatial resolution in a 24 x 24cm field of view, a slice thickness of 2mm, and zero slice gap. For DT images, TE = 84.4ms and TR = 16,000ms with the diffusion gradient encoding in 32 directions at b = 1,000 s/mm^2^ and an additional measurement without the diffusion gradient (b = 0 s/mm^2^). Data were recorded with a 128 x 128 spatial resolution in a 24 x 24cm field of view. A total of 50 sections were obtained with a slice thickness of 2.6mm and zero slice gap.

### T2w segmentations using ITK-SNAP

**Fig 1:**
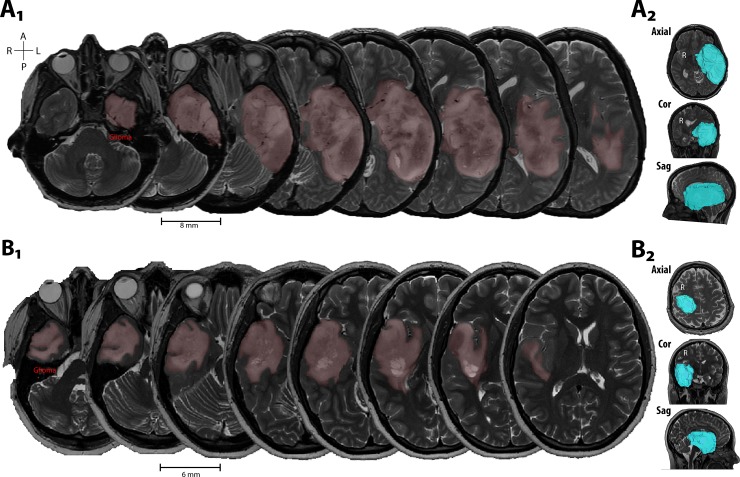
Examples of lesion volume segmentation and 3D rendering. (**A**, **B**) Axial T2w image sets from two patients with glioma. Manually segmented regions of hyperintensity displayed in light red. Side panels show 3D rendering of segmented lesion volume (cyan) in the axial (Ax), coronal (Cor), and sagittal (Sag) planes.

Preoperative T2w images were linearly registered to its respective DT images using DSI Studio (http://dsi-studio.labsolver.org) [31]. For all cases, the diseased tissue region (“lesion”) was manually segmented using ITK-SNAP [32] and registered, preoperative T2w scans acquired at most two weeks prior to surgery. T2w hyperintensities were used to define the lesion volume as the T2w hyperintensity identifies edema. The peritumoral edema volumes are expected to include the tumor volume as well [33]; see Fig 1 for example segmentations. Final segmentations were verified by a neurosurgeon (DRO).

### White matter tract processing

All processing steps were conducted using DSI Studio. The diffusion data were reconstructed using q-space diffeomorphic reconstruction [31] to obtain the spin distribution function [34]. A diffusion sampling length ratio of 1.25 was used. Restricted diffusion was quantified using restricted diffusion imaging [35]. The b-table was checked by an automatic quality control routine to ensure its accuracy [36]. Diffusion coefficient (AD, RD, MD, FA) maps were extracted for each case and used in the along-tract-analysis detailed below.

### Hemispheric Analysis

The Automated Anatomical Labeling Atlas (AAL) was used to autosegment left (L) and right (R) hemisphere regions of interest (ROI)[37]. Fiber tracking for the tumor hemisphere was determined by defining the hemisphere ipsilateral to the tumor as an ROI, and the contralateral hemisphere as a region of avoidance (ROA). For fiber tracking in the non-tumor hemisphere, the ROI and ROA are swapped: defining the hemisphere contralateral to the tumor as an ROI and the hemisphere ipsilateral to the tumor as a ROA. A deterministic fiber tracking algorithm [38] was applied using a whole-brain seeding region. The FA threshold was automatically set at 0.15. The angular threshold was 55 degrees. The step size was randomly selected from 1 voxel (i.e., 1 mm). The fiber trajectories were smoothed by averaging the propagation direction with a percentage of the previous direction. The percentage was 50%. Tracks with length shorter than 30 or longer than 300 mm were discarded. A total of 50000 seeds were placed (mean and standard deviation for track derivations across subjects was 48679.38 ± 1117.01). For all tract voxels AD, RD, MD, and FA values were extracted. Diffusion parameter values derived from the fibers seeded from the ipsilesional and contralesional hemispheres were compared using the shift function [39–41] which compares deciles of each distribution using the Harrell-Davis [42] quantile estimator to derive the difference between quartiles of group 1 and quartiles of group 2 as a function of one group of quartiles ($shift\;function=\frac{P1q-P2q}{P1q}$) with the *q*th quantile corresponding to the *P*th group (*P* = 1, 2) and controls for multiple comparisons by calculating the 95% confidence intervals of decile differences with a bootstrap (100 iterations; 200 samples per iteration) estimation of the standard error. Decile differences in which the confidence interval does not overlap with zero indicate decile locations within the distribution that are likely significantly different.

### Targeted white matter bundle analysis

The John Hopkins University White Matter (JHU-WM) [43,44] atlas was used to define the corticospinal tract (CST) and superior longitudinal fasciculus (SLF) white matter tract ROIs in both hemispheres. Fiber tracking was done using specific recipes of ROIs and ROAs: [1] Contralesional (C), ROI—contralesional hemisphere WM bundle (CST or SLF); ROA—ipsilesional hemisphere. [2] Ipsilesional (I), ROI—ipsilesional hemisphere WM bundle; ROA—contralesional hemisphere. [3] Ipsilesional Exclusive (IE), ROI—ipsilesional hemisphere WM bundle; ROA—contralesional hemisphere and tumor segmentation. [4] Ipsilesional Inclusive (II), ROI—ipsilesional hemisphere WM bundle and tumor segmentation; ROA—contralesional hemisphere. For all fiber tracking recipes, tracking parameters were identical to those described in the previous section. For all tract voxels, diffusion coefficients were extracted from their diffusion maps. The diffusion coefficients were normalized to their minimum and maximum values, thereby spanning a range between 0 and 1.

### Volumetric analysis

Tumor volume (cm^3^) was calculated for each case by counting the voxels contained in the segmentations created using ITK-SNAP multiplied by the voxel dimensions. Whole-brain volume was calculated from intracranial volumes composed of summating total gray and white matter volumes extracted from volBrain (http://volbrain.upv.es), an automated MRI brain volumetry pipeline [45,46]. For each case, tumor volume was normalized (tumor volume/ brain volume x 100 = % tumor volume) and correlated to normalized difference in diffusion coefficient (FA, MD, etc.). Normalized difference in each diffusion coefficient was calculated using:

$$\frac{D_{I}-D_{C}}{D_{avg}}x\;100$$ (1)

where *D* refers to the diffusion coefficient of interest, *I* refers to the ipsilesional hemisphere, *C* refers to the contralesional hemisphere, and *avg* indicates the average diffusion value derived from all tracts. To determine whether the area of the tumor was linearly related to average hemispheric diffusion estimates, a proxy for global white matter quality, Pearson correlations were used to compare the relationship between the normalized diffusion coefficient differences and percent tumor volumes.

### Along-tract-analysis

For CST fibers extracted using the JHU-WM ROI seed-based approach described earlier, we computed the mean tract length across subjects based on C, I, IE, or II categories. Then, using the mean tract length, we resampled the x, y, and z coordinates of each fiber to have the standard mean length using spline interpolation and decimation. FA and MD were extracted from the associated diffusion maps at the resampled positions and normalized to the mean FA and MD (respectively) of the contralesional hemisphere for each patient. The minimum Euclidean distance between the CST fiber voxels on the ipsilesional hemisphere and the lesion volume boundary was determined. The lesion volume was flipped onto the contralesional hemisphere and used for comparison of tract diffusion behavior as a within-subject control hemisphere.

## Results

In all patients, the affected tissue was manually segmented by outlining regions of hyperintensity on T2-weighted images and reviewed by a neurosurgeon. Fig 1 shows example segmentations of the lesion volume overlaid on axial T2-weighted images from two patients (Fig 1A: Grade 4 Glioblastoma; Fig 1B: Grade 2 Diffuse Astrocytoma).

**Fig 2:**
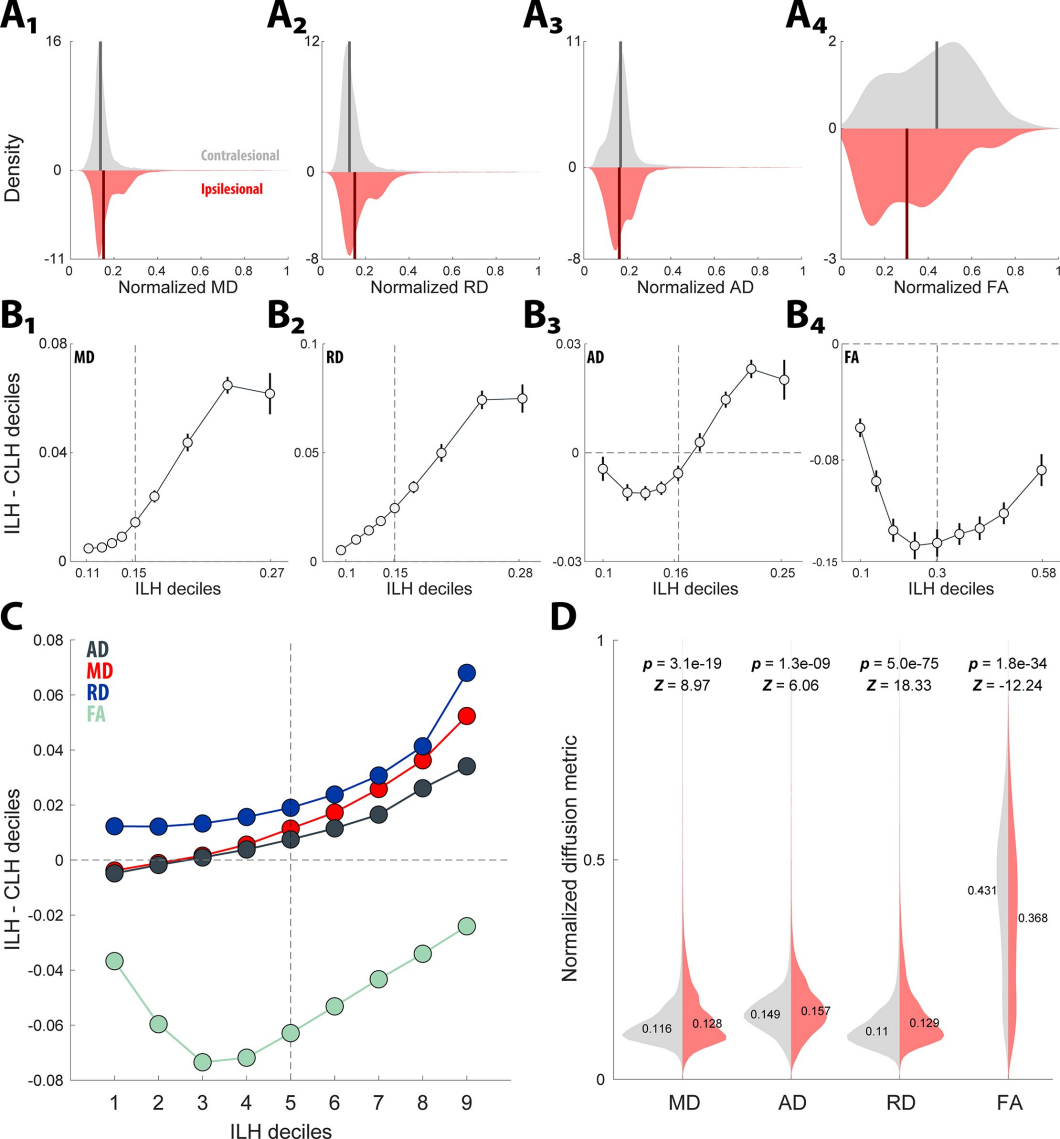
Hemispheric analysis of glioma impact on white matter. (**A**_1-4_). Hemispheric differences in the diffusion coefficient distributions (_1–4_; MD, RD, AD, FA, respectively) depicted as mirrored probability distributions (red indicates ipsilesional and gray represents contralesional; solid vertical line represents the median) for a representative case. (**B**_1-4_) A shift function analysis was used to compare differences over the entire distribution, by comparing the offsets of estimated deciles between the distributions (ILH = ipsilesional hemisphere, CLH = contralesional hemisphere). **B**_1-2_, shift function curves indicate that for both the MD, RD distributions there is a positive shift for both tails (more pronounced for the right tail), suggesting that at all segments of the distribution those values are higher for the ipsilesional side. **B**_3_, the shift function curve for AD shows a negative shift up to the median and then a steeper positive shift in the right tail of the distribution, indicating that likely the positive values in the right tail dominate the distribution and suggest an overall positive skew. **B**_4_, shows a dramatic negative shift function curve for the FA, indicating that the ipsilesional hemisphere is composed of lower values along the distribution compared to the contralesional hemisphere. (**C**) Group data (n = 13) for the shift function analysis strongly replicates the representative case in A&B. (**D**) Non-parametric t-tests (Wilcoxon rank sum) applied to the hemispheric distributions for each diffusion coefficient resulted in significant differences supporting the shift function findings: the ipsilesional hemisphere exhibited higher MD (Z = 8.97, p = 3.1e-19), higher AD (Z = 6.06, p = 1.3e-9), higher RD (Z = 18.33, z = 5.03–75) and lower FA (Z = -12.24, p = 1.8e-34) compared to the contralesional hemisphere.

Averaged hemispheric tract-based diffusion coefficients were analyzed between the ipsilesional and contralesional hemispheres. Fig 2A1-4 compares the diffusion coefficient distributions (AD, RD, MD, and FA) between the ipsilesional and contralesional hemispheres for a representative case (red identifies ipsilesional and gray indicates contralesional; the solid line demarcates the median). A shift function, which computes 95% confidence intervals for the difference in decile offsets to compare overall shape differences between the distributions, was utilized. Negative and positive offsets from 0, with confidence interval boundaries that do not intersect 0, indicate decile locations between the distributions that are statistically significantly different. Fig 2B1-4 shows that boot-strapped derivations of confidence interval boundaries for each subtracted distribution decile in Fig 2A1-4, show that all four diffusion coefficients differ between the hemispheres. Furthermore, the shift function results obtained from a single case (Fig 2A&2B), were representative of the population under study (Fig 2C). Finally, in Fig 2D, statistical analysis (Wilcoxon rank-sum test) of downsampled (by a factor of 50) tract-based diffusion coefficient distributions replicated the shift function analysis: the ipsilesional hemisphere exhibited higher MD (Z = 8.97, p = 3.1e-19), higher AD (Z = 6.06, p = 1.3e-9), higher RD (Z = 18.33, z = 5.03–75) and lower FA (Z = -12.24, p = 1.8e-34) compared to the contralesional hemisphere.

**Fig 3:**
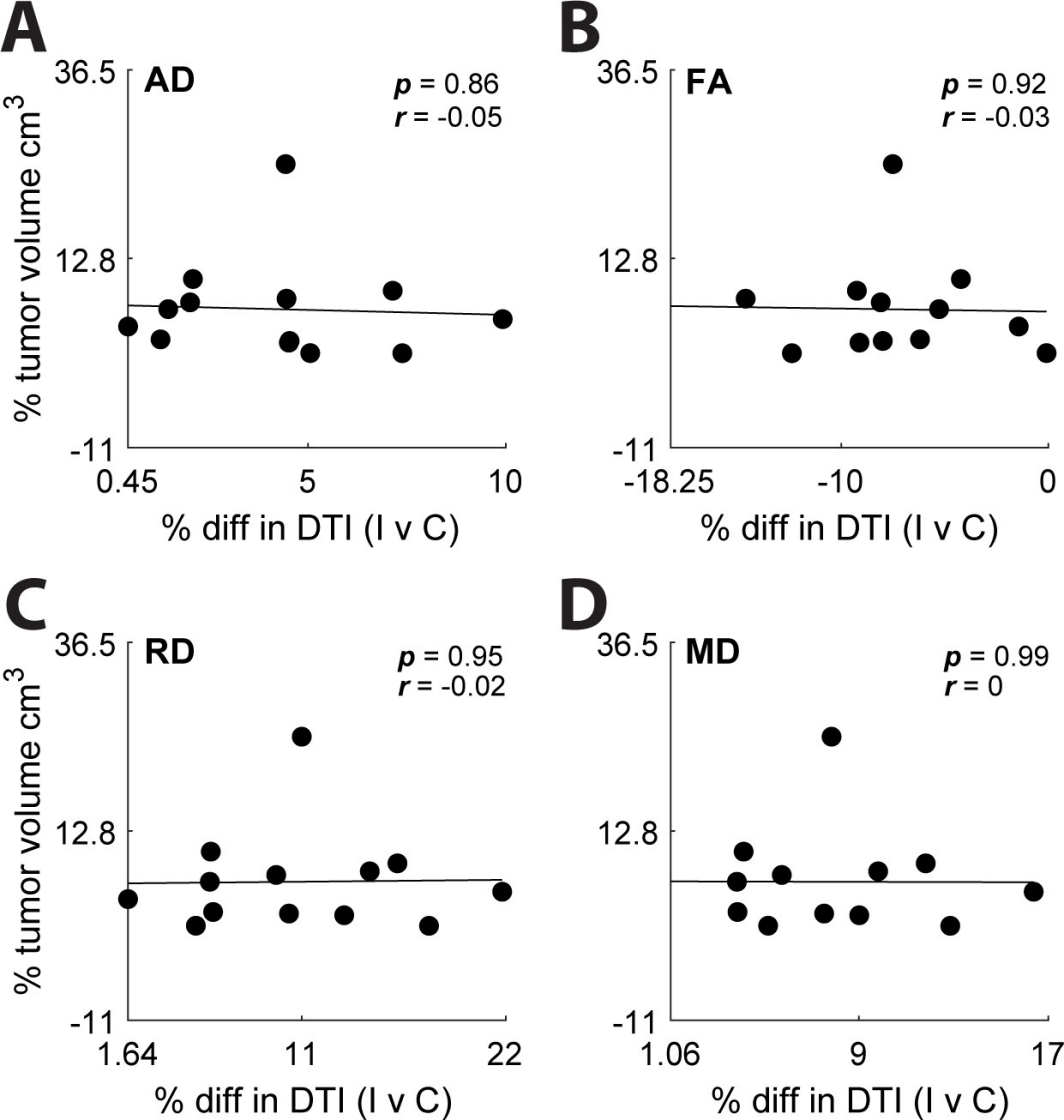
Volumetric analysis of glioma impact on white matter. (**A**: AD, **B**: FA, **C**: RD, **D**: MD). Percent volume of hemisphere occupied by tumor (tumor volume/tumor hemisphere volume) was plotted against each diffusion metric. No significant correlations were observed.

To determine whether the size of lesion contributed to the hemispheric difference in diffusion coefficient representation we assessed the relationship between the relative size of the lesion volume and the difference in hemispheric diffusion (Fig 3). AD, FA, and RD exhibited non-significant, linear correlations; with Pearson’s correlation coefficient equal to -0.05, -0.03, and 0.02, respectively. MD exhibited no linear correlation with Pearson’s correlation coefficient equal to 0.

**Fig 4:**
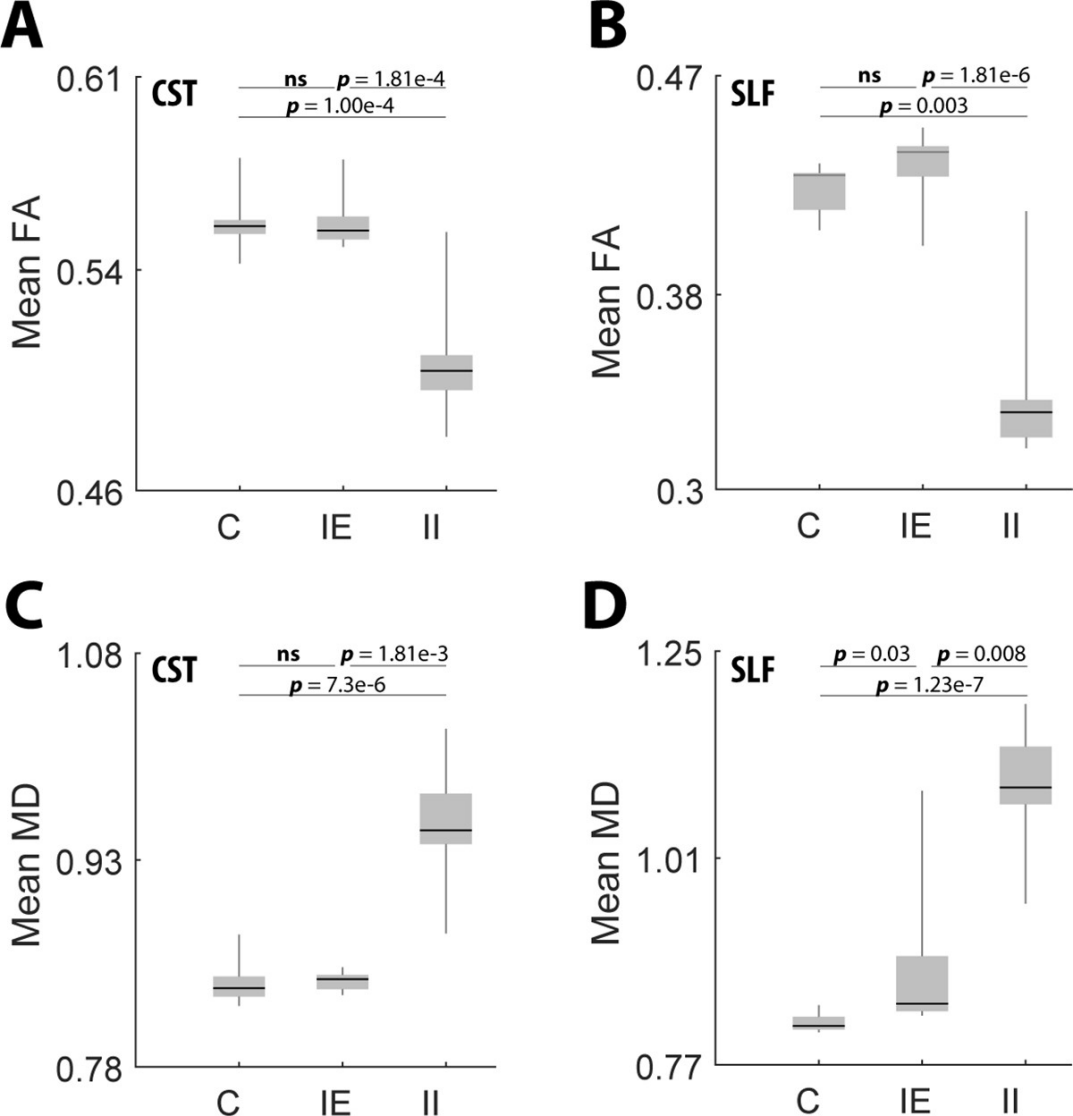
Mean diffusion coefficients along tractography varies when crossing the lesion volume. (**A**-**D**) Boxplots of mean diffusion coefficients along the CST and SLF grouped by C, IE, and II. P values for comparisons between groupings were calculated using post-hoc Dunn’s test analysis. Bars indicate the interquartile range.

Boxplots were generated to illustrate the distribution of FA and MD between the three experimental groups (C, IE, and II) for the CSTs and SLFs of all patients (Fig 4). Comparisons between the experimental group and diffusion coefficient for the isolated tracts were conducted using a Kruskal-Wallis H-test (a non-parametric ANOVA). Post-hoc Dunn’s test analysis was conducted for comparisons between experimental groups if H-test reached significance (p<0.05). For the CST, the Kruskal-Wallis H-test showed significant difference in distribution for FA and MD (FA_CST_, H(2) = 8.46, p = 0.0145; MD_CST_, H(3) = 9.557, 0.0084). The post-hoc analysis for the CST showed that II tracts had a significant decrease in FA and significant increase in MD compared to the C and IE tracts (FA_CST_: C v II p = 1.005 e-4; IE v II p = 1.81 e-4; MD_CST_: C v II p = 7.3 e-6; IE v II p = 1.81 e-3). No significant differences were seen in FA or MD of the CST between C v IE (FA_CST_: C v IE p = 0.708; MD_CST_: C v IE p = 0.68). For the SLF, the Kruskal-Wallis H-test showed significant differences in distributions of FA and MD (FA_SLF_, H(2) = 6.233, p = 0.044; MD_SLF_, H(2) = 19.004, p = 7.46E-5). The post-hoc analysis for the SLF showed that II tracts had a significant decrease in FA and significant increase in MD compared to the C and IE tracts (FA_SLF_: C v II p = 0.003; IE v II p = 1.81 e-6; MD_SLF_: C v II p = 1.23 e-7; IE v II p = 0.008). A significant increase was also observed in SLF IE MD compared to C (MD_SLF_: C v IE p = 0.03). No significant differences were seen in SLF FA between C v IE (FA_SLF_: C v IE p = 0.702).

**Fig 5:**
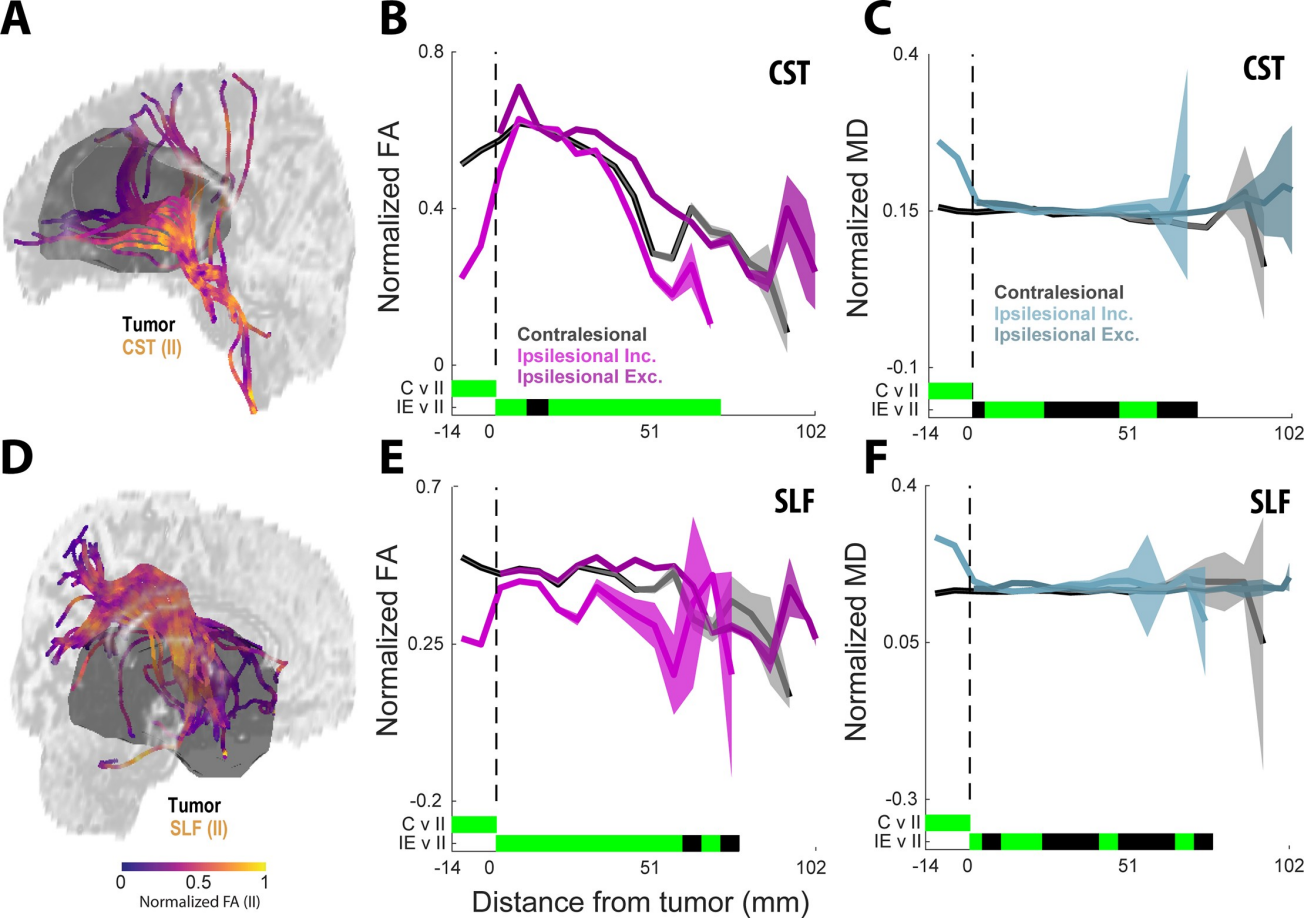
Diffusion coefficients along tractography influenced by proximity to lesion volume. (**A**) An example showing normalized FA variation with proximity to lesion volume is depicted for the CST. (**B**) Normalized diffusion coefficients of FA C, IE, and II groups plotted against distance from lesion volume boundary (mm), respectively. Negative values indicate tract locations within the lesion volume. The solid lines represent diffusion means at binned distances from lesion volume boundary, the shaded areas represent 95% confidence intervals (CI). Locations of overlapping CI are represented by the bars at the bottom of the plots, green indicates non-overlapping CI and black indicates overlapping CI. (**C**) Same as in **B**, but for MD. (D) An example showing normalized FA variation with proximity to lesion volume is depicted for the SLF. (**E**, **F**) Same as in (**B, C**), but summarizing across group data for SLF.

We sought to investigate the influence of the proximity of the lesion on the CST by inspecting changes to FA and MD with distance (Fig 5). An example of normalized FA variation, for both CST and SLF, with proximity to lesion volume is depicted in Fig 5A and 5D. In Fig 5B and 5C, the CST FA and MD for C, IE, and II groups were plotted against distance from lesion volume boundary (mm). The solid lines represent diffusion means at binned distances from lesion volume boundary, the shaded areas represent 95% confidence intervals, and green bars on the x-axis represent distances with non-overlapping confidence intervals. Negative x-axis values indicate tract locations within the lesion volume. The largest differences were observed within the lesion volume for both FA (Fig 5B) and MD (Fig 5C). Specifically, a sharp decrease in FA of the II tracts within the lesion boundary compared to the C tracts was observed. Meanwhile, a sharp increase in MD of the II tracts within the lesion boundary compared to the C tracts was observed. While MD stabilizes shortly beyond the lesion volume, FA fluctuations across the three groups continue at distances 50mm away from the lesion volume boundary. The values at the furthest distances from the lesion volume demonstrate higher variances due to fewer values at these locations and are predictably more variable.

## Discussion

Non-invasive, imaging-based evaluation of brain tumor infiltration and its impact on surrounding neural tissue is critical for diagnosing, treating, and monitoring the effects of tumor progression. Applications of diffusion imaging and DTI fiber tractography have been studied in the surgical treatment of brain tumor, including preoperative resection surgery planning [10,47,48], intraoperative assessment of functional boundaries correlated with cortical mapping [9,49–51], prediction of post-resection functional outcomes [52], and imaging-based extraction of histopathological features [51,53]. The work described in this study specifically extends the use of DTI fiber tractography for preoperative assessment of brain tumor impact on white matter. We used a within-subject experimental design (i.e., ipsilesional vs contralesional hemisphere comparison) to assess two major fiber bundles relevant to surgical resection, in a spatially normalized analysis (i.e., along-tract-analysis; [23]) of diffusion coefficients. In general, our results indicate that the impact of glioma on white matter is primarily localized to regions of the fiber bundles which pass through the tumor area (Fig 5), which is consistent with previous findings [51].

In our sample of subjects with glioma localized to a single hemisphere (Table 1), our initial analysis compared the contralesional hemisphere to the experimental ipsilesional hemisphere to determine the hemispheric impact of tumor on standard diffusion coefficients. We observed a significant increase in RD, AD, and MD and a significant decrease in FA in the ipsilesional hemisphere (Fig 2). This is indicative of higher water diffusion radially (RD), and axially (AD), less water restriction (MD), and weaker directionality (FA). This observation of glioma induced relative change in diffusion coefficients has been seen in previous studies, providing evidence for DTI’s consistency [18,24,54]. Aside from AD, the remaining three diffusion coefficients confer with animal and human studies that have correlated these parameters with histological quantification of myelin and axon degradation.

**Table 1:** Clinical information on patients used in this study.

Sex	Age	Hemisphere	Type	Grade	Lobe	T2-weighted hyper-intensity volume (cm^3^)
M	38	R	Anaplastic oligodendroglioma	3	Parietal	33.48
M	56	R	GBM	4	Frontal	14.37
M	22	L	GBM	4	Frontal	49.92
M	35	L	Anaplastic oligodendroglioma	3	Parietal	5.93
M	20	R	Oligodendroglioma	2	Frontoparietal	4.88
M	54	R	GBM	4	Frontotemporal	53.43
F	34	L	Oligodendroglioma	2	Frontal	41.33
F	23	R	Diffuse astrocytoma	2	Temporal	60.05
M	30	R	GBM	2	Frontal	16.21
F	73	L	GBM	4	Parietal	14.24
M	56	R	GBM	4	Parietal	27.85
F	42	L	GBM	4	Temporal	145.20
F	34	R	GBM	4	Frontal	45.92

Regarding AD, there is conflicting evidence in the literature regarding whether an increase or decrease in AD is consistent with the interpretation of AD as a reflection of axonal damage. Decreases in AD have been associated with axonal swelling and neurofilament dysfunction [17,54,55] in a mouse model. However, to date, the strongest evidence of the relationship between AD and axonal integrity in human neuroimaging studies, particularly in conditions associated with neurodegeneration, have reported increases in both RD and AD, including Huntington’s disease [56–59], Alzheimer’s disease [60–62], Parkinson’s disease [63] and Freidreich’s ataxia [64]. Consistent with these neuroimaging studies in neurodegenerative disorders, our finding of an increase in AD likely indicates increased axonal degradation.

Microstructural changes observed in neural tissue due to the presence of tumor cells are caused by infiltration, displacement, and/or destruction of the brain parenchyma (Jellison et al. 2004). In this study, we did not directly measure tract displacement, since we did not use a normal control for comparison. However, previous studies have demonstrated that the degree of mechanical displacement of brain tissue caused by mass effect is reflected by a change in diffusion coefficients [65]. To control for the relative difference in sampled voxels between the two hemispheres and to assess whether tumor mass correlated with any of the measured diffusion coefficients, we compared the lesion volume, as a fraction of total intracranial volume, with the percent difference in diffusion coefficient between the hemispheres (Fig 3). No correlation was found between volume and change in the diffusion coefficient, suggesting that the decrease in white matter structural integrity is not related to the affected tissue volume.

Hemispheric impact results next led to the focus on the spatially localized impact of tumor on white matter fiber bundles relevant to eloquent territories mapped during resection surgeries. As in our previous work (Ormond et al., 2017), we focused on the SLF and CST, which are associated with motor and language functions, respectively (Glasser and Rilling, 2008, Kamada et al., 2005). With this analysis, we observed that the impact of glioma on the measured diffusion coefficients (MD and FA) was highly localized to tracts that traversed the tumor region; only MD measured in SLF exhibited a significant difference between the ipsilesional exclusive tracts (tracts that did not traverse the tumor region) and the contralesional (control) hemisphere. In all comparisons, ipsilesional exclusive tracts were significantly different from the ipsilesional inclusive tracts (Fig 4). These are indicative of decreased microstructural integrity limited only to the tracts which cross the tumor area.

In the final analysis, we sought to identify whether the tracts within the fiber bundle traversing the tumor region (i.e., ipsilesional inclusive tracts), characterized by changes in diffusion coefficients consistent with white matter degradation (Fig 5), were either affected along the length of their tract or the change in diffusion signal was delimited by the proximity to the tumor. To answer this question, we modified an existing analysis, Along-Tract-Analysis (Colby et al., 2012), to allow for group level comparisons. Consistent with previous findings (Bucci and Staldheaur), across all subjects for both CST and SLF, we found that fiber coordinates located within and adjacent to the tumor region expressed diffusion coefficient values consistent with degradation when compared to coordinates along the same tract located more distal from the tumor (Fig 5). These findings indicate that the tumor impact on white matter structure appears confined to areas within and directly adjacent to the tumor.

Our findings support the ability for DTI to provide insight into the microstructural degradation caused by white matter tracts interacting directly with the tumor bulk and edema. Hemispheric AD, RD, MD, and FA coefficients demonstrated sensitivity to the presence of a lesion. Moreover, no relationship between these diffusion coefficients and the size of the lesion volume could be detected. Upon further investigation, the impact of the lesion volume on the primary diffusion coefficients FA and MD was primarily contained within the tracts that crossed the lesion volume. Finally, the influence of the lesion on the tracts passing through it appeared to be contained at the distance within or near the volume itself. Altogether, our study demonstrates the impact of glioma on tractography proximal to the site of the lesion.

## Limitations

This study comes with several limitations which should be improved upon in future experiments. To use a within-subject experimental design to compare diffusion imaging data, we included only those subjects with unilaterally localized glioma, which limits the generalizability of the results. This design was selected for two reasons: 1) development of an individualized approach to applying DTI analyses for preoperative surgical planning, and 2) avoid comparison to normal brain which could increase the likelihood of uninformative differences. Further, the along-tract-analysis employed in this study comparing inclusive and exclusive tracts from the ipsilesional hemisphere could be applied to whole-brain tractography in the context of midline crossing gliomas. Similar to previous studies, we derived tumor boundaries from T2w images, which has the inherent issue of confounding tumor, edema, and peritumoral regions (Stadlhear, Provenzale, Lu et al, 2014). However, as indicated in Fig 5, diffusion coefficient values (FA and MD) within and near the border of the tumor appear relatively stable, so whether the rim of the tumor region was composed of a combination of edema or tumor did not appear to affect the coefficient. For future studies, since both edematous and neoplastic tissue regions are believed to influence diffusion coefficients, one way to alleviate this issue would be to use higher resolution tensor estimations such as HARDI or Q-Ball, which help to resolve known issues concerning regions with complex diffusion patterns. Additionally, our method for identifying white matter bundles was intended to increase consistency and objectivity across subjects. However, as with any atlas-based ROI approach, the computed bundle for some cases may be significantly displaced due to the presence of the tumor. Finally, although our findings on the ipsilesional hemisphere regarding diffusion coefficients are consistent with compromised white matter, we must acknowledge that open questions remain regarding whether these parameters truly reflect aspects of axon or myelin degeneration.
